# Oscillatory dynamics as the coordination layer of the organism: waves, Markov blankets, and the virtual space of cognition

**DOI:** 10.3389/fnins.2026.1836602

**Published:** 2026-07-03

**Authors:** Thomas Dylan Daniel

**Affiliations:** Department of Psychological Sciences, Texas Tech University, Lubbock, TX, United States

**Keywords:** coordination layer, ecological psychology, enactivism, higher order affordances, Markov blanket, oscillatory dynamics, virtual space of cognition

## Abstract

Neural oscillations are not a mechanism that implements cognition. We present a new theoretical framework through a synthesis of relevant literature that has emerged in recent years: metabolic activity in the body, including but not limited to neural tissue, gives rise to an oscillatory pattern that contains information accessible to individual cells. The resulting dynamical structure allows cognitive activity to map the body in fine detail, to perceive its surroundings, or to extend into representations of objects and possibilities never encountered in the world. This array of possibilities is enabled by the coordination of the body’s components, which imposes invariant structural regularities among them, in turn creating a moment-to-moment series of states shared across a distributed network of cells. Metabolic success involves ensuring adequate access to nutrition and waste removal for every cell and, when achieved, can give rise to a series of leaps manifested as increased access to complex higher-order affordances. The body’s metabolic activity yields observable coordination; however, the mental actions themselves are inscrutable, existing only within a virtual space that unfolds in the interplay of the constituents of a particular body. Access to advanced functions is categorical; this virtual space expands during development and contracts in response to reduced metabolic sufficiency. Markov blankets formalize this asymmetry: brain-scanning technology clarifies the substrate, but no amount of information about the substrate provides direct access to cognitive activity. Frequency bands of oscillation correspond to spatial scales of inter-blanket communication, with cross-frequency coupling carrying information up and down the nested hierarchy. Several clinical conditions—ME/CFS, Long Coronavirus Disease (COVID), cancer-related cognitive impairment, Alzheimer’s disease, and age-related decline—share a common upstream mechanism within this framework: cellular damage degrades the substrate, which contracts the space of accessible cognitive operations and produces the categorical incapacity patients report. The framework generates a testable prediction: aperiodic spectral flattening should temporally precede the loss of specific oscillatory peaks as the substrate degrades.

## 1 Introduction: the coordination layer

### 1.1 Coordination as organism-wide wave-mediated engagement

The standard picture in cognitive neuroscience locates cognition in the brain and treats the body as a source of inputs and constraints. However, bodily configuration constitutively determines cognitive accessibility: the organism’s current physiological state—its metabolic capacity, inflammatory status, arousal level—determines which cognitive operations are available at all, not merely how efficiently they run. The deeper move called for is an expansion of cognitive psychology from neuroscience to physiology more generally: the brain is one participant in an organism-wide coordination, not a standalone computer receiving physiological inputs.

Damasio saw this first, or at least most clearly from within neuroscience. The somatic marker hypothesis (Damasio, 1994) demonstrated that bodily states are not peripheral inputs to rational cognition but constitutive participants in it—patients with ventromedial prefrontal damage who lost access to somatic signals did not merely feel less, they reasoned worse. *The Feeling of What Happens* (Damasio, 1999) extended this to consciousness itself: the organism’s continuous mapping of its own body provides the proto-self from which all higher-order cognition emerges. By *Self Comes to Mind* (Damasio, 2010), the argument was fully organismic—the self is constructed from body maps, and the brain’s cognitive operations are inseparable from the body they regulate. Damasio and Carvalho (2013) formalized feelings as homeostatic representations that integrate body-state information into cognitive processing. The present theory builds on this foundation: the means by which bodily states shape cognitive accessibility is the coordination layer, the oscillatory dynamics that connect cells throughout the brain, as well as the body.

Every cell in a multicellular organism participates in electrochemical signaling. Gap junctions create signaling networks across tissues—networks that store and maintain bioelectric patterns that serve as set-points for morphogenetic control, functionally analogous to memory and error correction. Whether they perform computation in the richer sense that neural networks do remains an open question. Ion channels maintain bioelectric gradients that encode cellular state (Hille, 2001; Levin, 2022). Paracrine signaling propagates chemical information through local neighborhoods (Singh and Harris, 2005). Neural transmission, the fastest channel, is a specialization of this pre-existing electrochemical coordination, not an invention of a fundamentally different kind of process (Jékely et al., 2015). Levin and Dennett’s “cognition all the way down” is the claim that the same computational principles operate from cells to brains: cells store target states, detect deviations, compute corrections, and navigate toward goals through novel paths. The nervous system is, in Levin’s framing, a “speed optimization” for pre-existing cellular competencies. McMillen and Levin (2024) formalize this as collective intelligence operating across scales and substrates, a framework that the present theory makes mechanistically specific through the oscillatory coordination layer.

Neural oscillations are this coordination layer operating at speed. When an alpha wave propagates through cortical tissue, each neuron it encounters either responds—transducing the signal, modifying it with local information, propagating the modified signal onward—or fails to respond. The responding neuron contributes its state to the organism-wide signal: the wave carries context from higher-order Markov blankets; the cell contributes its local condition; the resulting signal carries both. This wave-cell interaction is the source of information in the coordination layer. It is where bottom-up sensory states in the blanket formalism gain their content, which becomes relevant at the organism-wide level after initiating a local response to events that manifest at a meaningfully different level of scale.

The coordination is not limited to the brain. Cardiac cells communicate via electrical waves with conduction velocities and timing constraints that parallel neural oscillatory dynamics at slower timescales (Glass, 2001), relying on the same phosphocreatine buffering system, in which the PCr/ATP ratio predicts organ-level coordination failure (Neubauer et al., 1997). The enteric nervous system, with 500 million neurons, maintains its own oscillatory coordination (Furness, 2012). Immune cells form transient signaling networks through cytokine waves (Oyler-Yaniv et al., 2017). Bioelectric gradients across non-neural tissues store and propagate morphogenetic information (Levin, 2022). All of these are instances of the same process: wave-mediated inter-blanket communication, operating at different speeds and spatial scales.

What we call “cognition” in the narrow sense—thought, planning, and reasoning—arises atop this coordination when it operates through the fast, deeply nested, richly recurrent neural hierarchy. However, cardiac coordination is continuous with neural coordination, and the immune coordination is continuous with both. There is no sharp line where “mere biology” ends and “real cognition” begins, only a continuous gradient of speed, hierarchical depth, recurrence, and addressability.

### 1.2 The formal structure of inter-blanket communication

The coordination layer’s operation requires a precise account of what inter-blanket communication is, how it is identified, and what is being quantified. The Markov blanket framework (Friston, 2013; Kirchhoff et al., 2018; Friston et al., 2021) provides the formalism.

A system state is partitioned as x = (*ψ*, s, a, *μ*), where μ denotes internal states, ψ external states, and the blanket b = (s, a) comprises sensory states s (inward-facing) and active states a (outward-facing). The defining property is conditional independence of internal and external states given the blanket: μ ⊥ ψ | b. This property is not peculiar to biological or cognitive systems. It is the formal condition for a persisting, distinguishable region to exist: wherever a partition between inside and outside supports p(μ, ψ | b) = p(μ | b) · p(ψ | b), that partition constitutes a Markov blanket. Cells, organs, organisms, thermostat-regulated rooms, vortices in a stream, and sealed economies all instantiate this structure. The framework is general; its neuroscientific application is one case among many.

The virtual space of cognition is the internal perspective of a particular body within its context, as observed from the inside of the partition. The substrate of organism-wide oscillatory coordination is what the partitioning formalism captures from the outside. The substrate determines the shape of the virtual space, which operations are accessible, with what fidelity, across how many blanket scales. The substrate does not determine the content that flows through the virtual space, because content is generated and inhabited internally and cannot be derived from an outside-facing description. The study does not claim to fully explain cognition. It claims that the substrate places the limits on which cognitive operations are accessible to a given organism at a given moment, and that those limits and their dynamics are what the rest of this study investigates empirically. Though this research does not claim to solve the hard problem of consciousness, it observes that the Markov blanket brackets the interiority of the subject at different levels of analysis. These interior relations are self-similar across layers, and phenomenology is one facet of this interior.

The supervenience of the virtual space on the substrate is one-to-many. A given cognitive operation can be realized by multiple configurations of the coordination layer—different combinations of cells participating at different frequencies across different regions can produce the same macro coordination state. The cognitive operation does not control the substrate from outside. It traces a specific trajectory through the degenerate configurations available to it, and that trajectory is the path the substrate takes. In early Alzheimer’s disease, Mlinarič et al. (2026) observe that global aperiodic activity, averaged across all electrodes, shows no significant difference from controls. However, locally, posterior regions show aperiodic flattening, whereas frontal and temporal regions show aperiodic steepening. Simpler accounts (uniform cellular dropout, global inflammation, global metabolic decline) all predict global aperiodic change; none predicts the opposite-direction regional reorganization with preserved global signature. The pattern is what one would expect if cognitive operations supervene on a degenerate substrate (Edelman and Gally, 2001). Disease progression involves the system shifting among realizers of the same macro states while those macro states remain available. The cognitive trajectory persists; the local realization reorganizes.

“Inter-blanket communication” refers to two structurally distinct processes. Horizontal communication is the flow of information between blanketed systems at the same scale, where the active states of one system (a₁) become the sensory states of another (s₂). The physical channel varies by scale: gap junctions and exocytosis between adjacent cells, bloodstream-borne hormones between distant cells, vagal and endocrine signaling between organs, vocalization and gesture between organisms, messengers and broadcasts between groups. Vertical communication is information flow between nested blankets at different scales, where level-n blanket states become part of level-(n + 1) internal states, so that large-scale patterns modulate small-scale dynamics (top-down) while aggregated small-scale activity generates macroscopic patterns (bottom-up). Oscillatory dynamics operate in both directions simultaneously: a traveling wave is a large-scale pattern that modulates each cell it encounters while being sustained by the aggregated responses of those same cells. This is what justifies the focus of the study on oscillations: not that they are the only channel of inter-blanket communication, but that they are the distinctive channel that carries information up and down the nested hierarchy.

The formalism yields a corollary worth stating explicitly, as it is often left implicit in the FEP literature. Perception, the classical process by which external information becomes an internal state, is formally the transit *ψ* → s → *μ* through the inward-facing half of the blanket. Action is the complementary transit μ → a → ψ through the outward-facing half. Perception and action are therefore not two independent cognitive faculties to be explained separately; they are the two halves of any nonequilibrium system’s engagement with its environment, defined by the structure of its blanket. What differs between sensory modalities (vision, olfaction, proprioception) is the physical implementation of the inward channel; what differs between action modalities (speech, reach, pheromone release) is the physical implementation of the outward channel. The formal structure is invariant. In this framing, the coordination layer’s moment-to-moment function is to maintain and reconfigure the inter-blanket channels through which perception and action occur.

Inter-blanket information flow is measurable using domain-general information-theoretic quantities: mutual information I(μ₁; μ₂) across two systems, conditional mutual information I(μ₁; μ₂ | b₁, b₂) that tests blanket validity (approximately zero for valid blankets), transfer entropy T(μ₁ → μ₂) for directional flow, and partial information decomposition for multi-system integration (Schreiber, 2000; Williams and Beer, 2010). These measures apply to time-series data from any partitioned physical system. For oscillatory systems, each has a standard implementation: mutual information between oscillatory systems corresponds to phase-locking value, coherence, or debiased weighted phase-lag index (Lachaux et al., 1999; Vinck et al., 2011). Directional flow corresponds to directed PLV or phase transfer entropy; cross-scale flow is captured by cross-frequency coupling, including phase-amplitude coupling (Tort et al., 2010) and cross-frequency PLV. The theoretical claims of this study concern the phenomenon of inter-blanket information flow; the cited measures are the current instruments available to index it.

Markov blankets are identified by finding partitions whose conditional-independence property holds to within measurement tolerance. The procedure varies by system. For systems with physical boundaries, cells with membranes, organs with capsules, and organisms with skin, the blanket is given structurally, and the conditional-independence property is verified where relevant (Palacios et al., 2020). For systems with only functional partitions, the blanket is induced from the connectivity structure via community detection or clustering (Bassett and Sporns, 2017), with candidate blanket states at cluster boundaries and the conditional-independence test applied to verify. For oscillatory systems, this procedure has a band-specific variant: within-band coupling networks identify candidate blanket-bounded subsystems at the spatial scale characteristic of each frequency, and cross-frequency coupling identifies vertical communication between such subsystems. Hipólito et al. (2021) provided a five-scale implementation for neural data spanning single neurons through brain-wide networks. Friston et al. (2021) renormalization-group treatment of “parcels and particles” gives the recursive mathematical structure. Each of these is an instance of the same general identification approach applied to different physical systems.

The Markov blanket is the formal structure of any partitioned persistent system. Identifying blanket structure in such a system is descriptive and follows mathematically from the system’s existence as a distinguishable entity. Markov blanket partitions may be empirically observed, and the physical channels and level of scale of postulated systems of Markov blankets are empirically contestable. Two positions on the framework’s ontological status structure the recent literature, and the research resolves them dialectically before proceeding. Hipólito et al. (2021) applied the framework realistically: the partition is treated as a feature of the world to be discovered, with neural data interpreted as evidence for nested blanket structure across scales from single neurons to brain-wide networks.

Bruineberg et al. (2022) argued that this realist application conflates two distinct uses of the formalism—Pearl blankets are epistemic tools that summarize conditional independence in a probabilistic model, whereas Friston blankets are metaphysical boundaries posited as features of physical systems—and that ontological conclusions cannot be derived from epistemic instruments without further argument. Andrews (2021) put the same point compactly: the math is not the territory.

The *Formal Dialectics* framework (Daniel, 2018) offers a resolution. The partitioning we observe empirically—cell membranes, organ capsules, network communities, organism-environment boundaries—is real in the simple sense that it is what is encountered in the wild. The mathematics describes the structure of that observed partitioning, so the formal apparatus has descriptive purchase rather than being merely instrumental. The Bruineberg critique is preserved as well. Ontological conclusions about what lies inside the partition do not follow from the formalism, since it characterizes the partition from the outside. Its defining property is the decoupling of the inside of the Markov blanket from the outside. Therefore, Markov blankets are ideal for measuring the oscillatory coordination layer. Nevertheless, they cannot provide direct access to the internal experience of the organism. This is why cognition is theorized as occurring in a virtual space. The activity of the body at the metabolic level is observable but not entirely predictable due to the involvement of a metastable virtual cognitive pattern that partly drives events.

The specific claim is that in biological organisms, fast inter-blanket communication at scales relevant to moment-to-moment cognition is implemented by oscillatory dynamics at bands corresponding (centrally, not exclusively) to different blanket scales. The claim is falsifiable. If the conditional-independence property fails for the oscillatory clusters the theory identifies as blankets, the claim is wrong, and the central empirical argument requires revision. The general Markov blanket framework is unaffected by such a failure.

### 1.3 The cell is the minimal unit

The minimal unit of the organism-wide coordination is not a neuron computing a function. It is a cell encountering a wave and participating to a greater or lesser degree:

1) Effective participation—the cell’s ion channels respond to the incoming signal, its membrane potential changes, it fires or modulates its activity, and the modified signal propagates to its neighbors. The cell’s response adds its local state (metabolic condition, receptor occupancy, recent activation history) to the organism-wide signal. This is participation in the coordination layer. Crucially, the *degree* of participation varies continuously: spike-timing precision, firing probability per cycle, burst versus single spike, and subthreshold membrane engagement all modulate the cell’s contribution to the coordination pattern (Gutkin et al., 2005). Below critical coupling thresholds, oscillatory participation ceases entirely—a phenomenon known as oscillator death (Ermentrout and Kopell, 1990). Hippocampal place cells, for instance, systematically shift their firing phase within the theta cycle—precise phase carries information (O’Keefe and Recce, 1993). Participation is graded, not binary.
2) Effective exclusion—the cell fails to respond effectively, either because it lacks the metabolic resources to maintain the ion gradients required for transduction (the Na+/K + -ATPase pump consuming 55–75% of neuronal cellular ATP is the molecular mechanism of participation), because it is not currently coupled to the relevant wave frequency, or because it has lost the capacity to transduce the signal at all (damage, senescence, death). The cell contributes nothing or negligibly. The organism-wide coordination loses that node’s input.

The oscillatory cycle defines an *effective gating boundary*: on each cycle, communication windows open and close, and a cell either contributes meaningfully during that window or does not (Palmigiano et al., 2017). This gating is the discrete structure within which graded participation operates. Across billions of cells simultaneously, at multiple frequencies, through nested blanket levels, the aggregate pattern of graded participation produces the full complexity of cognitive life, and no single cell “thinks.” The thought is a property of the virtual space that emerges atop the coordination pattern. The virtual space of cognition is structurally emergent from the substrate of coordination patterns in which cells are currently participating, at which frequencies, and with what fidelity.

Buzsáki’s complementary framework (Buzsáki, 2010, 2015, 2019) emphasizes that oscillations organize *cell assemblies and sequences*—not individual cells deciding to “join the beat” but populations whose coordinated activation is structured by oscillatory timing. The coordination-layer theory and the assembly/sequence framework are compatible: assemblies are the units that oscillatory coordination organizes, and the coordination pattern across assemblies at multiple frequencies constitutes the virtual space.

The coordination layer’s spatial dynamics operate primarily through traveling waves rather than standing waves. Cortical oscillations propagate across neural tissue as traveling waves, generated by recurrent circuits and axonal conduction delays, transiently modulating spiking and excitability as they pass (Muller et al., 2018). Theta and alpha traveling waves in the human neocortex propagate at 0.25–0.75 m/s, with their propagation characteristics correlated to task performance (Zhang et al., 2018). This is not incidental to the theory—it is what the “cell encountering a wave” means physically. The wave moves through tissue; cells encounter it sequentially, not simultaneously.

The traveling-wave nature of oscillatory coordination has a specific computational consequence for the theory. Standing waves create periods when all neurons in a network are simultaneously in a low-excitability phase—the coordination layer would go periodically offline. Traveling waves ensure that a subset of neurons is always in a high-excitability phase, maintaining continuous coordination while still providing phase-dependent gating locally (Bhattacharya et al., 2022). Prefrontal oscillations during working memory organize as traveling waves, a property essential for maintaining persistent representations across oscillatory cycles. A coordination layer constituted by traveling waves never fully switches off and continuously shifts its zone of active participation through space.

The directionality of traveling waves adds a dimension that the frequency-band hierarchy alone does not capture. Alamia and VanRullen (2019) showed that backward-propagating alpha waves dominate during rest and forward-propagating waves during visual stimulation, corresponding to the direction of predictive versus error-driven inter-blanket communication. (Extensions of this finding come predominantly from Alamia, VanRullen, and collaborators; truly independent replication of the predictive coding interpretation remains limited, and alternative mechanisms—recurrent circuits, coupled oscillators—can generate similar traveling wave patterns.) Structural connectivity gradients in the human connectome shape traveling wave direction and frequency gradients (Koller et al., 2024), linking biophysical affordances to the spatial dynamics of coordination: cytoarchitecture and connectivity determine not only which oscillatory patterns a region can sustain but also the direction in which coordination flows through it.

More broadly, the coordination layer operates through a structural network whose topology constrains which coordination patterns are possible. Network neuroscience (Bassett and Sporns, 2017) has established that brain networks exhibit small-world topology, rich-club organization, and modular structure. These properties determine the efficiency, robustness, and repertoire of oscillatory synchronization patterns the network can sustain. Hub regions, which participate in the densest coordination traffic, are disproportionately vulnerable to neurological disease, a prediction the coordination-layer theory shares with the network neuroscience literature. The structural network provides the hardware through which the coordination layer operates; oscillatory dynamics provide the coordination patterns that the hardware supports. Neither account is complete without the other.

## 2 The architecture of the coordination layer

### 2.1 Frequency bands as blanket scales

If waves are inter-blanket communication, different frequency bands should correspond to communication at different scales of the nested Markov blanket hierarchy. The correspondence is structural, not metaphorical:

**Table 1:** 

Band	Dominant blanket scale	Communication function	Dynamics	Notable exceptions
Delta (1–4 Hz)	Organism-environment boundary	Homeostatic and motivational coordination between the organism and its surroundings (Harmony, 2013)	Seconds-to-minutes; state transitions	Local prefrontal delta during working memory (Harmony, 2013)
Theta (4–8 Hz)	Large-scale network coalitions	Coordination across organ-level blankets; assembly of distributed operations (Hasselmo et al., 2002; Kawasaki et al., 2010)	Hundreds of ms; working memory, navigation	
Alpha (8–12 Hz)	Network/organ-level blankets	Maintenance of coherent communication within and between major neural subsystems (Knyazev et al., 2011; Sadaghiani et al., 2010)	~100 ms cycles; DMN maintenance, sensory gating	Global alpha synchronization during working memory (Palva and Palva, 2007, 2011)
Beta (13–30 Hz)	Regional/circuit-level blankets	Stabilization and active content reactivation within inter-blanket configuration (Spitzer and Haegens, 2017; Lundqvist et al., 2016)	Tens of ms; status quo, content clearing	
Gamma (30–100 + Hz)	Local circuit/cellular blankets	Fine-grained cell-to-circuit communication; content-level information origination (Fries, 2005; Buzsáki and Draguhn, 2004)	Milliseconds; local computation, feature binding	Attention-dependent long-range gamma coherence (Gregoriou et al., 2009)

The mapping reflects the *dominant* spatial scale at which each band operates—grounded in the biophysical inverse relationship between oscillation frequency and spatial extent of the synchronized network (Buzsáki and Draguhn, 2004)—not an exclusive assignment. Gamma is generated locally but can synchronize across distances through inter-areal coherence; alpha operates primarily at the network scale but can coordinate globally. The table describes central tendencies, not strict partitions.

Cross-frequency coupling, the well-documented phenomenon where the phase of slower oscillations modulates the amplitude of faster oscillations, is what nested Markov blanket communication looks like in oscillatory terms. When alpha phase modulates gamma amplitude, a network-level blanket constrains the communication dynamics of local-circuit blankets within it. When the theta phase organizes alpha-gamma coupling sequences, a coalition-level blanket coordinates the activity of its constituent network-level blankets. Importantly, CFC is not strictly unidirectional: gamma bursts can reset theta phase (Lisman, 2005; Belluscio et al., 2012). This bidirectionality *enriches* the nested blanket interpretation: bidirectional CFC corresponds to bidirectional inter-blanket communication via active and sensory states, where lower-order blankets not only receive constraints from higher-order blankets but also propagate prediction errors upward through the hierarchy. A methodological caveat is essential: CFC measurement remains deeply contested, with non-sinusoidal waveform shapes generating spectral harmonics that can mimic genuine coupling (Cole and Voytek, 2017), and stringent controls have eliminated previously reported instances of theta-gamma phase coupling (Scheffer-Teixeira and Tort, 2016; Aru et al., 2015). The present theory does not depend on any particular CFC metric but rather on the biophysical phenomenon of cross-scale oscillatory interaction in which slow oscillations modulate membrane potential and gate fast oscillatory dynamics. CFC is one measurement window among several, alongside within-frequency coherence, traveling waves, and phase resetting.

This mapping generates a specific prediction about Kawasaki et al.'s (2010) finding that theta supported distant synchronization during working memory manipulation, while alpha supported local synchronization during retention. Under the coordination-layer framing, manipulation (assembling novel configurations) requires coalition-level blanket coordination (theta), whereas retention (maintaining an established configuration) requires network-level blanket maintenance (alpha). Different cognitive operations require coordination at different blanket scales, each indexed by a distinct frequency band. This is not a complication to be explained; it is the core structure.

### 2.2 The aperiodic substrate

Standard spectral analysis conflates two distinct components of the neural power spectrum: periodic oscillations, peaks rising above the background, and the aperiodic 1/f slope that constitutes the spectral background itself. Donoghue et al. (2020) demonstrated that these components can be parameterized separately, and that many historical findings attributed to “oscillatory” changes may reflect shifts in the aperiodic exponent. This is a methodological concern for the entire field of oscillatory neuroscience, including the findings cited in this study. Several of the alpha power changes documented in aging, sleep deprivation, and clinical populations were measured using methods that do not perform this decomposition. Until the specific findings this theory relies upon are re-analyzed with periodic/aperiodic separation, there is uncertainty about the relative contributions of each component.

However, not all reported research results are equally vulnerable. Causal evidence, alpha-frequency tACS strengthening DMN connectivity (Clancy et al., 2022), and 10 Hz stimulation enhance creativity. While 40 Hz does not drive oscillations at a specific periodic frequency, it observes specific effects that cannot be explained by aperiodic shifts. Phase-based measures (phase-amplitude coupling, inter-regional phase coherence, phase-dependent gating of spike timing) index temporal relationships between periodic cycles and are structurally distinct from aperiodic spectral shape. The most vulnerable claims are those resting solely on spectral power in a single band measured without decomposition, including portions of the clinical evidence in Section IV.

A deeper question is whether the aperiodic slope and the oscillatory peaks are independent at all, and they are not. They are different aspects of the same behavior by the same cellular populations. The aperiodic exponent reflects aggregate excitatory/inhibitory balance in neural populations (Gao et al., 2017). E/I balance is not independent of oscillatory coordination. It is the biophysical condition that determines whether neural tissue can sustain coherent oscillations at all (Buzsáki and Wang, 2012). Inhibition-dominant networks (steeper slope) produce sharper oscillatory peaks and more precise temporal coordination; excitation-dominant networks (flatter slope) produce noisier, less structured activity with degraded oscillatory precision.

In coordination-layer terms, the aperiodic decomposition and the oscillatory decomposition are two windows to the same cellular activity. The Na+/K + -ATPase pump maintains the intracellular ionic gradients that actuate both the aperiodic substrate and the periodic oscillatory coordination. When metabolic capacity degrades through aging, inflammation, and mitochondrial dysfunction, the E/I balance shifts, the aperiodic slope flattens, and the body’s capacity to sustain structured coordination diminishes. The aperiodic change is not a rival to the oscillatory account. It is a different view of the same cellular machinery that undergirds the oscillatory mechanics.

This reframing generates a specific prediction not made by the aperiodic literature alone: aperiodic flattening should temporally precede the loss of specific oscillatory peaks, because the local substrate can degrade before the global coordination patterns that include it collapse. Voytek et al. (2015) demonstrated aperiodic flattening with age and showed that this flattening statistically mediated age-related working memory impairment. The prediction is regional, not global. Flattening should be expected in regions where coordination-relevant cells are dropping out, and may coexist with opposite-direction changes in regions sustaining compensatory load (Mlinarič et al., 2026). Whole-brain averaging across electrodes can wash out these regional effects, hiding local problems, which may explain some of the inconsistencies in the aperiodic Alzheimer’s disease literature. Kopčanová et al. (2024) found no aperiodic differences between AD patients and controls when using whole-brain averages, despite clear changes in periodic components. This finding is consistent with the regional qualification above and further points to a virtual space with functional outcomes that can be reached via alternative means (degeneracy) when specific neural infrastructure becomes unavailable.

The coordination-layer theory interprets regional aperiodic flattening as the cells that enact the metabolic support operation that undergirds cognition becoming progressively less capable of sustaining the structured oscillatory activity that constitutes cognitive accessibility. The closest existing longitudinal study by Finley et al. (2024) showed that resting IAPF and aperiodic exponent jointly predict cognitive decline over 10 years. However, since the study measured EEG at only one time point, it was not sufficient to test the prediction that aperiodic changes temporally precede oscillatory degradation in individuals. Such an observation would be expected if age-related cognitive impairment has its roots in metabolic decline: the higher-order act of attempting to remember tends to persist despite occasional or persistent trouble recalling specific details. In Alzheimer’s disease in particular, regional coordination decline can be partially compensated by other regions taking on additional load, which preserves cognitive accessibility until the compensating regions themselves fail.

The theory acknowledges that aperiodic broadband activity carries cognitive and clinical information (Lu et al., 2024; He et al., 2010). It does not claim that all functionally relevant neural dynamics are periodic. The metabolic activity of the body is observable in the aperiodic substrate and the oscillations that arise atop this backdrop, as both phenomena are expressions of the same regional cellular metabolism at different scales.

### 2.3 Neuromodulation as slow-timescale coordination

Neuromodulators are chemicals diffused into the extracellular environment of brain tissue, shaping broad firing patterns among neural populations over a seconds-to-minutes timescale (Marder, 2012). Neuromodulation shapes the operational configuration of the coordination layer, but it does not entail the propagation of specific signals.

The coordination layer operates at multiple timescales simultaneously. The frequency-band hierarchy from delta through gamma captures the fast dynamics—milliseconds to seconds. However, the oscillatory mode the coordination layer occupies at any given moment is itself determined by a slower layer of coordination: neuromodulatory systems operating at seconds-to-minutes timescales through volume transmission and metabotropic receptor cascades.

Acetylcholine, dopamine, norepinephrine, and serotonin do not control the coordination layer from outside. They constitute inter-blanket communication at a slower temporal scale. Acetylcholine paces the hippocampal theta-phase encoding/retrieval switch; high cholinergic tone biases the system toward entorhinal-driven encoding at the theta peak, low cholinergic tone toward CA3-driven retrieval at theta trough (Hasselmo et al., 2002). Cholinergic blockade in humans disrupts both theta amplitude and phase alignment during encoding, with the magnitude of phase disruption predicting the degree of memory impairment (Gedankien et al., 2023). Acetylcholine does not “turn on” theta from outside; it modulates the coordination pattern’s parameters from within, shifting the balance between encoding and retrieval modes that the theta cycle organizes.

More broadly, neuromodulatory systems configure which oscillatory state the coordination layer occupies: acetylcholine and norepinephrine jointly regulate the integration of top-down predictions and bottom-up sensory evidence, with acetylcholine signaling expected uncertainty and norepinephrine signaling unexpected uncertainty (Yu and Dayan, 2005). Each neuromodulatory system modulates cross-frequency coupling in specific ways, shaping not only single-band power but the nested inter-band relationships that the theory identifies as inter-blanket communication (Weiss et al., 2023). Zhang et al. (2024) demonstrated that neuromodulators interact cooperatively and divergently across brain states, with their shifting configurations associated with distinct hippocampal oscillatory patterns during NREM sleep, REM sleep, and wakefulness.

In blanket terms, neuromodulatory systems operate at a higher level of the temporal hierarchy, constraining the operating parameters of the faster oscillatory coordination beneath them. There is no tuner outside the instrument.

### 2.4 Biophysical affordances

Canolty and Knight (2010) showed that cross-frequency coupling varies across cortical regions: parietal cortex has different temporal integration properties from the temporal cortex. If regional oscillatory profiles constitute biophysical affordances, properties of the local tissue that channel which coordination patterns are most easily sustained, then functional specialization is not Fodorian modularity but convergent channeling.

A cortical region’s intrinsic oscillatory properties (determined by cytoarchitecture, local inhibitory interneuron density, thalamocortical connectivity, and myelination patterns) determine which inter-blanket communication patterns it can most efficiently sustain. The “math/language partition” and similar functional dissociations are not hard-wired modules but reflect that different computations require different coordination patterns, and different regions afford different coordination patterns due to their biophysical properties.

The Gibsonian extension: just as a cliff affords falling-off for creatures of a certain size, a cortical region affords certain coordination patterns for networks with certain oscillatory profiles. The affordance exists in the coupling between function and tissue, not in either alone.

This extension has precedents in the embodied cognition literature. Bruineberg and Rietveld (2014) explicitly connected affordance responsiveness with metastable neurodynamics and the free energy principle, arguing that adequate engagement with affordances requires the kind of metastable coordination dynamics the present theory foregrounds. Djebbara et al. (2021) directly measured oscillatory correlates of architectural affordances, finding that alpha desynchronization covaries with the affordance properties of physical environments, providing empirical evidence that affordance perception is constitutively linked to oscillatory dynamics rather than merely implemented by them. Frequency gradient mapping across the cortical surface (Mahjoory et al., 2020) provides further empirical grounding: systematic spatial variation in dominant oscillatory frequency reflects cytoarchitectonic gradients, connecting the biophysical affordance concept to measurable tissue properties rather than relying solely on regional CFC variation.

## 3 Alpha: an entry point

### 3.1 The default mode network is the intrinsic manifold at the network-blanket scale

The controllosphere framework (Holroyd, 2025) distinguishes two processing regimes: the intrinsic manifold, where the system operates within configurations that are sustainable at baseline metabolic cost, and the controllosphere, where the dorsolateral prefrontal cortex pays continuous metabolic expenditure to hold the system in non-default configurations. The default mode network consists of the posterior cingulate cortex/precuneus, medial prefrontal cortex, bilateral angular gyri, and is the intrinsic manifold expressed in hemodynamic and oscillatory terms. The default mode network’s operations are the cognitive operations that abound when the system does not push into the controllosphere. Alpha is treated here as the best-documented entry point, not as “the” rhythm of cognition. It is the largest-amplitude scalp-recorded oscillation, has been studied since Berger’s work in the 1920s, and corresponds to the network-blanket scale that cognitive neuroscience most directly measures.

The causal coupling between alpha oscillations and DMN connectivity makes this identification empirically concrete. Clancy et al. (2022) showed that alpha-frequency transcranial alternating current stimulation over the occipitoparietal cortex augmented alpha oscillations and strengthened BOLD connectivity within the core DMN (PCC-mPFC); posterior-to-frontal alpha connectivity increases mediated the DMN enhancement, with no effects occurring outside alpha frequency or outside the DMN. Ma et al. (2025) extended this by showing that alpha-tACS tightened the dynamic temporal coupling between spontaneous alpha power fluctuations and DMN connectivity, establishing that alpha does not merely co-occur with the DMN but actively maintains its temporal coherence. MEG findings converge on this picture: DMN functional connectivity is expressed in both alpha and beta bands, with alpha-band amplitude envelope correlations providing the primary mechanism for long-range DMN connectivity (Brookes et al., 2011; Hillebrand et al., 2016), and Knyazev et al. (2011) found alpha to be the only frequency band with substantial spatial overlap with DMN topology.

DMN connectivity is not exclusively alpha-band. Hacker et al. (2017) found that theta bandlimited power correspondence was stronger in the DMN than alpha using electrocorticography, and Mantini et al. (2007) found the DMN’s electrophysiological signature spanned alpha and beta. This multi-frequency involvement is predicted by the coordination-layer framework: a network-level blanket communicates predominantly at its characteristic frequency (alpha), coordinates with higher-scale blankets (theta), and stabilizes internal configurations through lower-scale dynamics (beta). The causal specificity of alpha-frequency stimulation (Clancy et al., 2022; Ma et al., 2025) establishes alpha’s privileged role in DMN maintenance without requiring that it be the sole channel.

Under the coordination-layer framing, these findings establish that alpha-band inter-blanket communication is the dominant coordination channel of the intrinsic manifold at the network-blanket scale. The DMN is supported primarily by alpha-band coordination among its constituent region-level blankets, with beta and theta contributing to intra-network stabilization and cross-network integration, respectively. Causal tACS evidence shows that strengthening the alpha coordination channel strengthens the functional architecture because the dominant channel is the architecture’s backbone.

The metabolic logic is grounded in thermodynamics. The brain consumes approximately 20 watts, 20% of resting metabolism. Within the cortex, communication consumes 35 times more energy than computation: of ~4.94 watts total cortical expenditure, ~3.52 watts supports communication while only ~0.10 watts funds computation proper (Levy and Calvert, 2021; Notably, “computation” here is defined narrowly as postsynaptic ionotropic receptor activation). The Na+/K + -ATPase pump consumes 55–75% of total brain ATP. Fewer than 1% of cortical neurons can be substantially active concurrently (Lennie, 2003). Alpha-band coordination enforces this sparsity by maintaining tonic inhibition over most cortical regions; the alpha coordination pattern keeps the system within the metabolic budget of the intrinsic manifold.

### 3.2 Executive function is a coordination pattern, not a controller output

The homunculus problem, the infinite regress of controllers controlling controllers, has persisted because each attempt to decompose the “central executive” finds subfunctions that themselves require coordination. Miyake et al. (2000) decomposed the executive into inhibition, shifting, and updating; each subfunction still faces Fodor’s frame problem: how does inhibition “know” what to inhibit?

Within the coordination-layer framework, this question dissolves completely, not just at the alpha level but at all blanket scales simultaneously. Executive function is not the output of a controller region. It is the input to the virtual space written into the coordination layer to map onto the body. It is the detection and then enactment of mental possibilities. The homunculus is retired, but the body still refuses to reduce to biophysics.

The empirical evidence for this dissolution is strongest at the alpha-band/network-blanket scale. Sauseng et al. (2005) showed that under higher executive demands, frontal alpha short-range connectivity decreased, whereas fronto-parietal long-range coherence increased in the theta and upper alpha bands. Executive function shows up as a change in the coordination pattern, not as activation of a control region. Palva and Palva (2007, 2011) demonstrated that alpha-band synchronization networks carry a dual role: content-specific subnetworks in sensory areas maintain working memory contents while shared fronto-parietal subnetworks reflect the top-down executive network that selects the to-be-remembered visual contents. The communication-through-coherence framework (Fries, 2005, 2015) provides one candidate mechanism for these effects, with empirical support from selective gamma-band synchronization during attention in primate visual cortex (Bosman et al., 2012). However, Schneider et al. (2021) provided evidence that inter-areal coherence may be a consequence rather than a cause of communication, with oscillatory power changes better explaining cross-areal coupling. The coordination-layer framework is more general than CTC and compatible with multiple underlying mechanisms, including power-based communication, resonance (Hahn et al., 2014), and transient synchrony (Palmigiano et al., 2017). What matters for the present theory is that oscillatory dynamics gate inter-areal communication, not the specific mechanism by which they do so. The PFC’s role under this framing is as a metabolically expensive coordination hub, a region whose dense long-range connectivity and sustained firing capacity make it the primary node for maintaining non-default coordination patterns across blanket scales.

### 3.3 Predictive coding as inter-blanket communication dynamics

Alpha oscillations map onto the predictive coding architecture with a specificity that the coordination-layer framing explains. Alamia and VanRullen (2019) showed that a hierarchical predictive coding model with physiological communication delays (~12 ms) naturally generates alpha-band rhythms, with backward alpha traveling waves dominating during rest.

The coordination-layer theory adds the distinction that standard predictive coding lacks: the distinction between precision-weighting (continuous modulation within an established communication channel) and categorical accessibility (whether the communication channel exists at all). A region excluded from alpha-band coherence is outside the relevant network-level Markov blanket: categorically inaccessible, not merely downweighted.

## 4 Clinical convergence: coordination-layer degradation

Patients with cancer-related cognitive impairment (CRCI), ME/CFS, and Long Coronavirus Disease (COVID) report categorical incapacity—“I can’t think,” not “thinking is harder.” These are not “brain conditions” with peripheral metabolic complications. They are organism-wide coordination-layer degradations that manifest most visibly in cognition because cognitive operations require the widest-spanning, most metabolically expensive coordination patterns.

### 4.1 Metabolic coordination failure: ME/CFS

ME/CFS involves mitochondrial dysfunction, including reduced ATP production, impaired pyruvate dehydrogenase function, and a shift from oxidative phosphorylation to less efficient glycolysis (Fluge et al., 2016). Recent molecular work adds specificity: WASF3 disrupts mitochondrial respiratory supercomplex assembly, reducing Complex IV levels in skeletal muscle (Wang et al., 2023). Under the coordination-layer framing, cells throughout the body lose the metabolic capacity to participate in wave-mediated coordination. EEG evidence is broadly consistent but imprecise: the most reliable findings in ME/CFS are increased delta/theta and decreased beta power, with alpha changes inconsistent across studies (Zinn et al., 2018; Silva-Passadouro et al., 2024). Under the coordination-layer interpretation, multi-band disruption is expected to cause metabolic failure that degrades coordination capacity across the hierarchy, not at a single blanket scale. The coordination-layer theory predicts this broad spectral disruption; the finding does not uniquely confirm the theory. Preliminary MRS evidence suggests brain creatine metabolism is altered in ME/CFS (Godlewska et al., 2024), consistent with the metabolic substrate argument developed below.

Post-exertional malaise is the characteristic signature (Institute of Medicine, 2015) in which activity depletes ATP faster than mitochondria can regenerate it. Cells begin dropping out of the coordination layer. The crash is delayed because ATP depletion is cumulative, and the coordination layer has redundancy. But below a threshold of participation, the coordination pattern cannot sustain itself: the coordination loses critical mass and wide-spanning operations fail categorically.

The observation of hysteresis in neural systems (Kelso, 1995), in which it becomes harder to regain access than to lose it, reflects a fundamental property of coordination. Rebuilding coordination is more expensive than maintaining it, which is why recovery takes longer than depletion. This is a structural property of any coordination layer operating without reserve, not a feature unique to ME/CFS. The same hysteresis should appear in Long COVID, in CRCI recovery trajectories, and in the post-hospitalization decline pattern in early AD.

### 4.2 Inflammatory coordination disruption: long COVID

Inflammatory signals propagate through the same signaling channels that carry the coordination layer’s inter-blanket communication (Davis et al., 2023). They are noise in the channel. “Brain fog” is the phenomenology of a coordination layer operating with a degraded signal-to-noise ratio (Hampshire et al., 2024). The fog is not a metaphor; it is an accurate description of what degraded coordination precision feels like from inside. Consistent with the metabolic substrate argument, creatine supplementation improved patient- and clinician-reported fatigue outcomes in post-COVID patients (Slankamenac et al., 2023).

### 4.3 Cellular damage and coordination loss: CRCI

Chemotherapy damages cells throughout the body, not just tumor cells (Lange et al., 2019). Each damaged cell is a node lost from the coordination network. The construction machinery (novel problem-solving, cognitive flexibility) fails first because it requires the widest coordination span. Hardwired responses persist because they require only local coordination among intact circuits.

### 4.4 Alzheimer’s disease: progressive coordination-layer death

Babiloni et al. (2023) finding that reduced DMN gray matter volume in Alzheimer’s disease correlates with reduced alpha source activity in posterior DMN hubs documents coordination-layer death at the network-blanket scale. The order of cognitive decline in AD maps onto the coordination span: episodic memory (requiring the widest coordination) before procedural memory (local circuit coordination). Executive function before sensorimotor function. Novel problem-solving before habitual responses (Bäckman et al., 2005).

The progression of AD is the progressive contraction of the organism-wide coordination’s reach: first, the widest-spanning coordination fails, then progressively more local coordination, until eventually even basic local circuits lose coherence. Treatment-response evidence supports the dynamic: in mild cognitive impairment, levetiracetam reduces hippocampal hyperactivity and improves memory (Bakker et al., 2012), consistent with the prediction that compensation can be doing more harm than good once the substrate it is loading exceeds its capacity.

This account emerges from an analysis of what initially appeared to be a weakness in the regional aperiodic literature. Heterogeneous changes across different neurological regions during early AD progression explain why damage to metabolic capacity does not immediately result in the failure of the cognitive apparatus of the organism. This is also why diagnosis and intervention should begin with an assessment of cellular metabolic capacity: the metastability of higher-order patterns in the virtual space of cognition can obscure diagnostic measures, as these patterns may function normally despite substrate damage.

### 4.5 Compensation and collapse

The framework predicts an extended compensation dynamic that precedes the collapse of high-level functions. The tendency of tissue to maintain function despite damage explains the difficulty of observing direct aperiodic degradation prior to the complete collapse of the coordination layer, which accompanies noticeable impairment. The coordination layer theory predicts that healthy regions can maintain functionality only by entering the controllosphere to compensate for damage at an increased metabolic cost. Compensating regions show an *increased* aperiodic exponent (steeper slope), reflecting the increased inhibitory load required to stabilize the configuration to support higher-order functions. This is the pattern Mlinarič et al. (2026) observed in preclinical and prodromal Alzheimer’s disease: posterior parieto-occipital flattening of the degrading region, consistent with the well-established FDG-PET signature of posterior hypometabolism in early AD, accompanied by frontal/temporal steepening, the compensating regions absorbing the load.

Compensation operates on a metabolic budget that the compensating regions support without reserve. Eventually, failure occurs due to sustained load and lack of rest implied by the supporting role these tissues take on. When this occurs, the anterior aperiodic exponent shifts from steepening to flattening, and the compensated function becomes categorically unavailable. The experience an individual has during this transition is what families and clinicians describe as the sudden decline leading to hospitalization: the categorical drop that marks the failure of compensatory measures. This is not the onset of pathology, but rather the failure of the support systems that had previously maintained functionality in the face of adverse interstitial conditions.

The compensation dynamic generalizes to a broader degeneracy principle (Edelman and Gally, 2001): the coordination layer’s macro states are realized by multiple distinct configurations of local cellular activity. Cognitive operations are not tied to specific local circuits; they are tied to macro states that can be produced by different combinations of cells participating across different regions. Therefore, disease progression is not simply substrate degradation; it is the exhaustion of degenerate configurations. Categorical accessibility transitions occur when the last available configuration capable of realizing a macro state fails. This explains why patients with similar cognitive impairment show different underlying EEG patterns, why within-individual cognitive performance varies day-to-day, and why focal lesions can leave some operations preserved despite the apparent loss of a “responsible” region; the operation was never the property of that region, but of the macro state the region was contributing to.

### 4.6 The intervention hierarchy

The coordination-layer theory generates a structurally principled intervention hierarchy:

1) Cellular metabolic capacity first. Restore cells’ capacity to participate: mitochondrial support, inflammation reduction, pacing to prevent metabolic debt, senolytics. Creatine supplementation enhances both corticomotor excitability and cognitive performance under metabolic stress (Turner et al., 2015), consistent with the prediction that restoring the metabolic substrate restores coordination capacity.
2) Autonomic/organ coordination second. Optimize slower blanket-scale dynamics: autonomic regulation, exercise, sleep optimization.
3) Network/oscillatory coordination third. Once the substrate can sustain it: neurofeedback, tACS, meditation training.
4) Organism-environment coordination fourth. Psychotherapy, social support, and environmental modification.

The hierarchy respects the nesting: you cannot optimize network-level coordination if cells cannot sustain it. The ME/CFS push-crash dynamic occurs when interventions target the wrong level.

### 4.7 The PTSD and depression cases

PTSD is coordination-layer quenching: a rapid precision spike freezes the coordination pattern into a defensive configuration. Nicholson et al. (2023) double-blind RCT showed that alpha-desynchronizing neurofeedback produced 60% remission at 3-month follow-up, a promising preliminary finding that awaits independent replication. (The 60% figure would be unusually high; neurofeedback studies have historically faced concerns about inadequate controls and expectancy effects—Thibault et al., 2017, 2018.)

Depression involves both coordination rigidity and contraction of virtual space. The locked pattern AND the contracted space are both real: locking causes contraction, because a coordination layer stuck in one mode cannot assemble the diverse wave-cell interactions that wider coordination requires.

### 4.8 Senescence as a coordination-layer degradation

Cellular senescence anywhere in the body should impair cognitive function because senescent cells disrupt the organism-wide coordination layer in which the brain participates. Peripheral senescent cell burden should correlate with cognitive decline, even when the senescent cells are not neurons.

Senescent cells: (1) stop responding appropriately to intercellular signals, (2) emit disruptive signals via the SASP, and (3) recruit neighbors into dysfunction through paracrine senescence (Coppé et al., 2010; Nelson et al., 2012).

Preclinical evidence supports this prediction: clearing senescent cells in tau-dependent neurodegeneration models prevented cognitive decline (Bussian et al., 2018), and dasatinib plus quercetin improved hippocampal-dependent memory in aged mice (Ogrodnik et al., 2021). In humans, the SToMP-AD pilot clinical trial (Gonzales et al., 2022) demonstrated the safety and feasibility of senolytic therapy in early-stage Alzheimer’s disease but was not powered to assess cognitive endpoints. The preclinical case is strong; the human evidence is promising but preliminary.

### 4.9 Aging: a novel perspective

The coordination layer framework positions intercellular communication as a main driver of health. In aging, the coordination layer suffers as a result of general systemic disruption that is difficult to explain from the standpoint of traditional biological and/or psychological vantages. The result: the virtual space contracts with age. This observation is consistent with the pathophysiology literature and mainly consists of a synthesis of pre-existing ideas.

Taylor et al. (2026) have produced the most rigorous continuous lifespan chart of cortical functional connectivity organization to date (*n* = 3,556, 16 days to 100 years). This work shows that human brain functional connectivity (FC) changes in a distinct way, producing an inverted-U with gradient dispersion peaking at 13.8 years, SA and MR axes peaking in early adulthood, and all three steadily contracting afterward. Structure–function coupling steadily decreases throughout life, with the sharpest decline for transmodal axes. Ruuskanen et al. (2026) mapped the analogous lifespan trajectory of MEG-resolved oscillatory frequency-specific functional connectivity. The convergence is evident in the alpha-band FC decline associated with age, the beta-band FC following an inverted-U with midlife peak, and the delta/theta/gamma FC rising. Taylor et al. (2026) and Ruuskanen et al. (2026) together provide a view of this substrate contraction across the human lifespan, showing that cellular substrate degradation can be observed at different scales.

This manifests as cognitive decline, reduced physical coordination, immune dysregulation, impaired wound healing, and reduced homeostatic capacity. These are the *coordination-dependent* aging phenomena, and they share a mechanism. Not all aging is coordination-layer degradation; presbyopia, cochlear hair cell loss, photoaging, and cataract formation involve material and structural changes not primarily mediated by coordination failure. The “one phenomenon” claim applies to coordination-dependent aging, which is the majority of age-related functional decline.

### 4.10 Prediction

Interventions that restore cellular participation in the coordination layer should simultaneously improve function across all coordination-dependent domains. Senolytic therapies should improve cognition, not just tissue health. Exercise, which improves mitochondrial function, reduces inflammation, and enhances cellular signaling capacity across all tissue types, should have cognitive benefits disproportionate to its direct neural effects. It does (Erickson et al., 2011).

## 5 Discussion

Traditional neuroscience commits a category error by localizing cognition in the brain, treating a distributed coordination pattern as a property of one organ, and erroneously ascribing abstract operations to physiology. The body is not an input to cognition. The body is a participant in the same coordination layer as the brain. Coupling of the body to its environment is enabled by the dynamics of the coordination layer, without a centralized controller. Cognition emerges atop this dynamic structure; its content cannot be derived from a description of the substrate, no matter how complete.

Stoffregen and Wagman (2025) have produced an account of higher-order affordances that balances parsimony with elegance in effectively explaining behavior using non-reductive machinery. We suggest here that this account is directly relevant to the future of cognitive neuroscience, which must explain the brain in terms of the body’s behavior as well as the individual actions of its cells.

Process philosophy, the critique of treating processes as substances, provides the meta-theoretical framework (Nicholson, 2019). The central executive is not a distinct, independent entity in the PFC. Instead, executive function is the pattern of wave-mediated inter-blanket communication across the organism at a given moment, most observable in cortical tissue due to the properties of neural cells. This pattern has no fixed spatial address because it is a relationship among cells, not a property of any one of them.

The enactivist tradition provides the cognitive-science lineage for these commitments. Varela et al. (1991) argued that cognition is not internal representation but embodied action; Thompson (2004) extended this through the life-mind continuity thesis, grounding cognitive processes in the self-organizing dynamics of living systems. Chemero (2009) explicitly situated HKB coordination dynamics within radical embodied cognitive science; Aguilera et al. (2013) demonstrated that “cognitive behavior is the result of recurrent sensorimotor and brain oscillatory coordination at multiple scales,” arriving at a claim structurally identical to the present theory through the enactivist rather than the neuroscientific route. The coordination-layer theory’s specific contribution is not the insight that cognition is embodied, distributed, and dynamical, which belongs to the enactivist tradition. Rather, the contribution is grounded in a specific, empirically measurable substrate: the nested oscillatory hierarchy mapped to Markov blanket scales, with quantifiable predictions about when coordination succeeds or fails.

### 5.1 Objections

#### 5.1.1 The reification question

The most penetrating objection: “The coordination layer” may be a description mistaken for an explanation. Treating ‘the coordination layer’ as an ontological discovery rather than a descriptive convenience risks reifying a useful summary into a causal-explanatory entity. When we say “the coordination layer reorganizes experience during rest,” are we explaining something, or are we redescribing what individual neurons, synapses, and circuits do using a higher-level vocabulary that adds no mechanistic insight? If every claim about “the coordination layer” reduces without remainder to claims about specific neural populations, specific oscillatory dynamics, and specific metabolic processes, then “the coordination layer” is a convenient label, not an ontological discovery.

The defense: The theory’s claims about “the coordination layer” do reduce, but they reduce to claims about *measurable oscillatory dynamics*: power spectral density in specific bands, phase-amplitude coupling strength between specific frequency pairs, inter-regional coherence at specific frequencies, and the metabolic capacity to sustain these dynamics. These are quantifiable, manipulable, and falsifiable. The coordination layer is not a ghost in the machine; it is a shorthand for a specific set of oscillatory relationships that can be measured, perturbed, and predicted. The claim is that this set of relationships is not merely correlated with the coordination required to support cognition, but constitutive of it, and that claim is empirically testable through the bifurcation arguments outlined below.

The genuine risk of reification lies in the theory’s organism-wide claims. When the theory extends from well-documented neural oscillatory dynamics to cardiac coordination, immune signaling, and bioelectric morphogenesis, the evidence for constitutive (rather than merely causal) relationships thins considerably. The organism-wide coordination layer may be a productive metaphor that outruns its empirical warrant.

#### 5.1.2 The falsifiability question

What evidence would kill the theory? A framework that cannot specify its own failure conditions is not a scientific theory but a narrative. The coordination-layer theory commits to falsification criteria, while acknowledging that its scope is not all-inclusive. Section I establishes a dialectical resolution (Daniel, 2018) that delimits the ability of description with respect to the analysis of particular systems. This framework brackets off the internal relations that occur within a given Markov blanket, specifying that these are not available to full empirical verification. Thus, claims about what goes on within these partitions are unverifiable. However, the substrate that underwrites categorical accessibility is, in fact, available for investigation using empirical means. Therefore, falsifiability includes four criteria:

What would kill the theory:

1) Substrate–accessibility dissociation. Demonstrating that restoring oscillatory dynamics to their healthy configuration (correct frequencies, coupling strengths, coherence patterns) *fails* to restore the corresponding cognitive operations. If the substrate determines which cognitive operations are accessible, restoring the substrate to its healthy configuration must restore that accessibility. A clean dissociation would falsify the theory.
2) Cognitive improvement without oscillatory change. A pharmacological or behavioral intervention that demonstrably improves a specific cognitive operation without any detectable change in the oscillatory dynamics will disprove the theory.
3) Non-oscillatory complex cognition in biological systems. Discovering a biological system that exhibits complex, flexible, hierarchically organized cognition without oscillatory coordination dynamics. This would refute the substrate-accessibility claim by demonstrating that the same cognitive phenotype can be realized in a non-oscillatory substrate.
4) Blanket decomposition irrelevance. Demonstrating that the Markov blanket decomposition of oscillatory dynamics adds no predictive or explanatory power beyond simpler descriptions (e.g., regional activation levels, pairwise connectivity). If the blanket structure does no explanatory work, the theoretical apparatus is unnecessary.

#### 5.1.3 The anesthesia problem

The objection: Oscillations persist under general anesthesia, but cognition stops. If oscillatory dynamics constitute cognition, how can the dynamics persist while cognition vanishes? This appears to be a direct counterexample to the substrate accessibility claim.

The response: The substrate accessibility claim concerns *nested, multi-scale cross-frequency coupling with the right hierarchical structure*, not about single-band oscillatory power or the mere presence of CFC. Anesthesia does not merely reduce oscillatory activity—it fundamentally reorganizes it. General anesthetics characteristically: (1) *preserve or enhance* single-band oscillatory power, particularly in the alpha and delta ranges (propofol produces prominent frontal alpha; sevoflurane enhances delta); (2) *reorganize* cross-frequency coupling into abnormal patterns—Mukamel et al. (2014) found propofol induces a rapid transition between distinct CFC states, with phase-amplitude coupling inverting from the normal “trough-max” pattern to an abnormal “peak-max” pattern during deep unconsciousness, while Purdon et al. (2013) characterized the full electroencephalographic signatures of propofol-induced loss and recovery of consciousness, including novel slow-oscillation/alpha coupling that appears only under anesthesia; and (3) *fragment* long-range inter-regional coherence, disconnecting the coordination layer’s hierarchical organization into locally coupled but globally uncoordinated oscillators (Akeju and Brown, 2017; Mashour and Hudetz, 2018).

The distinction between CFC destruction and CFC reorganization is critical for the theory’s precision. Anesthesia does not eliminate cross-frequency coupling—it replaces the nested, hierarchically organized CFC that the theory identifies as constitutive with a different set of coupling relationships: locally maintained, phase-inverted, disconnected from the multi-scale architecture. The orchestra analogy is apt but must be stated precisely: anesthesia is not silence, and it is not even every section playing independently. The sections still maintain internal temporal relationships—CFC persists locally—but those relationships are inverted and disconnected from the larger score. The music vanishes not because the coupling stops but because the coupling loses its hierarchical structure. Oscillatory activity supports structured, nested, multi-scale coordination—the specific pattern of CFC, not its mere presence.

This is an empirically testable distinction: if the theory is correct, restoring the *hierarchical structure* of CFC under anesthesia (correct phase relationships, multi-scale nesting, long-range coherence) should restore at least partial cognitive function, even if total oscillatory power remains unchanged. Conversely, reorganizing CFC into non-hierarchical patterns while preserving its local presence should abolish cognition, which is precisely what anesthesia does.

#### 5.1.4 The developmental problem

The objection: Infant oscillatory profiles differ markedly from those of adults. Neonatal EEG is dominated by discontinuous activity, trace alternant patterns, and delta brushes. Mature alpha rhythms do not emerge until 3–4 months, and adult-like frequency profiles are not fully established until adolescence. If oscillatory coordination constitutes cognition, infant cognition should be impossible, or the mapping between specific oscillatory patterns and specific cognitive operations should hold across development. Neither appears to be the case.

The response via the gradient framework: The theory does not claim that a specific oscillatory profile constitutes cognition in general. It claims that the *available* oscillatory coordination determines the *available* cognitive operations. Infant cognition is real, but it is also profoundly different from adult cognition: slower, less deeply nested, less recurrent, and less flexibly reconfigurable. This is precisely what the coordination-layer theory predicts from the infant oscillatory profile.

Development, under this framing, IS the progressive elaboration of the coordination layer: the emergence of faster frequency bands (gamma maturation in the first year), the establishment of cross-frequency coupling (theta-gamma coupling developing through childhood), the myelination-dependent increase in long-range coherence (continuing through adolescence), and the metabolic maturation that supports sustained coordination (oxidative phosphorylation capacity increasing through childhood). Each developmental milestone in oscillatory dynamics corresponds to new cognitive capabilities: the emergence of alpha correlates with sustained attention; the maturation of theta-gamma coupling correlates with the development of working memory capacity; the establishment of long-range fronto-parietal coherence correlates with the emergence of executive function.

### 5.2 Theoretical considerations

Developmental EEG evidence broadly supports this correspondence, but the mapping is imprecise. The correlation between oscillatory maturation and cognitive development is well-documented (Saby and Marshall, 2012; Uhlhaas et al., 2010), but the evidence shows predominantly co-occurrence rather than oscillatory changes preceding cognitive milestones. A confound remains: structural development—myelination, GABAergic maturation, synaptic pruning—may independently drive both oscillatory and cognitive changes without a direct constitutive link between them. The developmental case is currently more consistent with the theory than hostile to it, but it does not yet provide strong confirmatory evidence.

#### 5.2.1 The circularity question

The theory defines cognition as coordination, observes that all cells coordinate, and concludes that all cells are cognitive. Formally valid but potentially trivial. The non-trivial defense: the definition is motivated by explanatory success, not arbitrary stipulation. If the expanded definition generates confirmed predictions that narrower definitions miss, it earns its keep.

A principled line against cognitive bloat: the four dimensions of variation—speed, hierarchical depth, recurrence, and addressability—provide gradient criteria. The autopoietic boundary provides an additional constraint: only self-producing, self-maintaining systems are candidates. This excludes rocks and crystals while including cells and organisms, and at some point, along the gradient, the concept of mind loses explanatory grip. However, that boundary is principled, not arbitrary, because it tracks the biophysical capacity for self-producing coordination rather than an observer’s judgment about complexity.

#### 5.2.2 The homunculus dissolution

The homunculus is retired—not by finding it in a smaller region, decomposing it into subprocesses, or declaring it unreal—but by identifying what it was always a placeholder for: the coordination layer itself. There is no controller because the virtual space’s geometry IS the control.

This dissolution has predecessors: Dennett (1991) multiple drafts, Blanke and Metzinger (2009) self-model theory, Friston’s active inference, and most directly, Kelso (1995, 2012, 2021) coordination dynamics. The coordination-layer theory’s contribution is to extend Kelso’s insight from motor coordination to cognition more generally and to ground it in the multi-scale blanket hierarchy.

The regress objection persists in a specific form: if frontal theta coordinates posterior alpha (Helfrich and Knight, 2016), what coordinates frontal theta? This question presupposes the controller architecture that the theory dissolves. Frontal theta does not control posterior alpha as a thermostat controls a heater. Both are aspects of the same multi-scale coordination pattern—the apparent directionality describes the pattern’s structure, not a causal chain requiring a first mover. In coordination dynamics (Kelso, 1995), the relative phase between coupled oscillators is not controlled by either oscillator but emerges as a property of their coupling. The regress terminates not in a smallest controller but in the recognition that there was never a controller to begin with, only a self-organizing coordination pattern whose current geometry determines what the system does next.

#### 5.2.3 The parsimony challenge

Most of the theory’s clinical predictions are also predicted by simpler, established models. An inflammation model and a metabolic model each predict the clinical phenomenology of CRCI, ME/CFS, and Long COVID without requiring the coordination-layer apparatus.

The coordination-layer theory must therefore foreground the predictions that distinguish it from accounts that focus solely on inflammation or metabolism. First, domain-specific co-improvement patterns: the inflammation model predicts approximately uniform improvement, whereas the coordination-layer theory predicts improvements that reflect the coordination architecture. Second, oscillatory specificity: peripheral senescent cells should impair cognition specifically by disrupting oscillatory dynamics, rather than by general inflammation. Third, categorical boundaries: performance distributions near coordination-threshold boundaries should be bimodal rather than normally distributed around a degraded mean. Until these distinguishing predictions are tested, the theory’s clinical implications, while consistent with the evidence, do not require the coordination-layer explanation.

## 6 Conclusion

Cognition is not implemented by the brain alone. Locating it there is a category error. Treating a distributed coordination pattern as a property of one organ is a sure way to miss the forest for the trees. The brain participates in cognition, but it does not house it. Instead, the brain is a center through which an oscillatory coordination layer passes electrochemical signals that enable trillions of cells to access relevant bodily information across tissues and timescales. The brain passes signals rapidly, recursively, and deeply, but it is still only one participant in a body-wide coordination.

Markov blankets provide an adequate description of this coordination from an external vantage. They describe the observable metabolic underpinnings of cognition while making space for the yet-unknown internal experience of the organism to fluctuate arbitrarily, including made-up or imaginary content alongside externally-driven inputs, without falling into reductive determinism or failing to account for observable relationships. The partition is real and can be approached mathematically, but it brackets the interior reality of the organism away from the observable exterior and acknowledges that there remains a component of cognition that substrate observation and mathematics cannot predict.

The architecture of the coordination layer is multi-band and nested. Frequency bands map onto communication scales, cross-frequency coupling implements the nested hierarchy, and aperiodic activity reads out the same cellular state through a different decomposition. Neuromodulation configures the operating mode at slow timescales, and regional cytoarchitecture channels influence the coordination patterns that each tissue sustains most effectively. The availability of coordination drives the flow of information through the body, and this flow of information is a prerequisite for the higher-order activity researchers refer to as cognition.

The same physiological coordination explains the shared phenotype of ME/CFS, Long COVID, cancer-related cognitive impairment, Alzheimer’s disease, and age-related cognitive decline. Each condition involves specific pathways of dysfunction that lead to the same consequences: individual cells drop out of the coordination layer, and complex operations, such as advanced cognition, become unavailable because the resource it requires is no longer accessible. This framework makes the testable prediction that aperiodic spectral flattening should temporally precede the loss of specific oscillatory peaks in individuals because the cellular substrate can degrade before the tissue becomes completely unable to sustain any such activity. Degeneracy enables damaged tissues to continue to function; the temporal order of decline the framework predicts is an aperiodic decline at first, then the loss of particular oscillatory peaks. If aperiodic flattening fails to precede oscillatory peak loss within the regions where coordination is degrading, the framework is wrong. This is the empirical commitment the framework stakes.

The homunculus is retired. The brain–body distinction is dismantled. Coordination remains a distributed activity carried out at the cellular level on an ongoing basis throughout the body. The ability of the organism to participate in advanced cognitive activity depends upon this oscillatory coordination, shifting the question from reference to the central executive to the coherence of the rhythms that emerge from innumerable individual actions and reactions.

## Data Availability

The original contributions presented in the article are included in the article/supplementary material; further inquiries can be directed to the corresponding author.

## References

[ref1] Aguilera M. Bedia M. G. Barandiaran X. E. (2013). The situated HKB model: how sensorimotor spatial coupling can alter oscillatory brain dynamics. Front. Comput. Neurosci. 7:117. doi: 10.3389/fncom.2013.00117, 23986692 PMC3750630

[ref2] Akeju O. Brown E. N. (2017). Neural oscillations demonstrate that general anesthesia and sedative states are neurophysiologically distinct from sleep. Curr. Opin. Neurobiol. 44, 178–185. doi: 10.1016/j.conb.2017.04.011, 28544930 PMC5520989

[ref3] Alamia A. VanRullen R. (2019). Alpha oscillations and traveling waves: signatures of predictive coding? PLoS Biol. 17:e3000487. doi: 10.1371/journal.pbio.3000487, 31581198 PMC6776260

[ref4] Andrews M. (2021). The math is not the territory: navigating the free energy principle. Biol. Philos. 36:30. doi: 10.1007/s10539-021-09807-0

[ref5] Aru J. Aru J. Priesemann V. Wibral M. Lana L. Pipa G. . (2015). Untangling cross-frequency coupling in neuroscience. Curr. Opin. Neurobiol. 31, 51–61. doi: 10.1016/j.conb.2014.08.002, 25212583

[ref6] Babiloni C. Lopez S. Noce G. Ferri R. Panerai S. Catania V. . (2023). Relationship between default mode network and resting-state electroencephalographic alpha rhythms in cognitively unimpaired seniors and patients with dementia due to Alzheimer’s disease. Cereb. Cortex 33, 10514–10527. doi: 10.1093/cercor/bhad363, 37615301 PMC10588004

[ref7] Bäckman L. Jones S. Berger A.-K. Laukka E. J. Small B. J. (2005). Cognitive impairment in preclinical Alzheimer’s disease: a meta-analysis. Neuropsychology 19, 520–531. doi: 10.1037/0894-4105.19.4.520 16060827

[ref8] Bakker A. Krauss G. L. Albert M. S. Speck C. L. Jones L. R. Stark C. E. . (2012). Reduction of hippocampal hyperactivity improves cognition in amnestic mild cognitive impairment. Neuron 74, 467–474. doi: 10.1016/j.neuron.2012.03.023, 22578498 PMC3351697

[ref9] Bassett D. S. Sporns O. (2017). Network neuroscience. Nat. Neurosci. 20, 353–364. doi: 10.1038/nn.4502, 28230844 PMC5485642

[ref10] Belluscio M. A. Mizuseki K. Schmidt R. Kempter R. Buzsáki G. (2012). Cross-frequency phase–phase coupling between theta and gamma oscillations in the hippocampus. J. Neurosci. 32, 423–435. doi: 10.1523/jneurosci.4122-11.2012, 22238079 PMC3293373

[ref11] Bhattacharya S. Brincat S. L. Lundqvist M. Miller E. K. (2022). Traveling waves in the prefrontal cortex during working memory. PLoS Comput. Biol. 18:e1009827. doi: 10.1371/journal.pcbi.1009827, 35089915 PMC8827486

[ref12] Blanke O. Metzinger T. (2009). Full-body illusions and minimal phenomenal selfhood. Trends Cogn. Sci. 13, 7–13. doi: 10.1016/j.tics.2008.10.003, 19058991

[ref13] Bosman C. A. Schoffelen J. M. Brunet N. Oostenveld R. Bastos A. M. Womelsdorf T. . (2012). Attentional stimulus selection through selective synchronization between monkey visual areas. Neuron 75, 875–888. doi: 10.1016/j.neuron.2012.06.037, 22958827 PMC3457649

[ref14] Brookes M. J. Woolrich M. Luckhoo H. Price D. Hale J. R. Stephenson M. C. . (2011). Investigating the electrophysiological basis of resting state networks using magnetoencephalography. PNAS 108, 16783–16788. doi: 10.1073/pnas.1112685108, 21930901 PMC3189080

[ref15] Bruineberg J. Dolega K. Dewhurst J. Baltieri M. (2022). The emperor’s new Markov blankets. Behav. Brain Sci. 45:e183. doi: 10.1017/s0140525x21002351, 34674782

[ref16] Bruineberg J. Rietveld E. (2014). Self-organization, free energy minimization, and optimal grip on a field of affordances. Front. Hum. Neurosci. 8:599. doi: 10.3389/fnhum.2014.00599, 25161615 PMC4130179

[ref17] Bussian T. J. Aziz A. Meyer C. F. Swenson B. L. van Deursen J. M. Baker D. J. (2018). Clearance of senescent glial cells prevents tau-dependent pathology and cognitive decline. Nature 562, 578–582. doi: 10.1038/s41586-018-0543-y, 30232451 PMC6206507

[ref18] Buzsáki G. (2010). Neural syntax: cell assemblies, synapsembles, and readers. Neuron 68, 362–385. doi: 10.1016/j.neuron.2010.09.023, 21040841 PMC3005627

[ref19] Buzsáki G. (2015). Hippocampal sharp wave-ripple: a cognitive biomarker for episodic memory and planning. Hippocampus 25, 1073–1188. doi: 10.1002/hipo.22488, 26135716 PMC4648295

[ref20] Buzsáki G. (2019). The Brain From Inside out. Oxford: Oxford University Press.

[ref21] Buzsáki G. Draguhn A. (2004). Neuronal oscillations in cortical networks. Science 304, 1926–1929. doi: 10.1126/science.1099745, 15218136

[ref22] Buzsáki G. Wang X.-J. (2012). Mechanisms of gamma oscillations. Annu. Rev. Neurosci. 35, 203–225. doi: 10.1146/annurev-neuro-062111-150444, 22443509 PMC4049541

[ref23] Canolty R. T. Knight R. T. (2010). The functional role of cross-frequency coupling. Trends Cogn. Sci. 14, 506–515. doi: 10.1016/j.tics.2010.09.001, 20932795 PMC3359652

[ref24] Chemero A. (2009). Radical Embodied Cognitive Science. Cambridge: MIT Press.

[ref25] Clancy K. J. Andrzejewski J. A. You Y. Rosenberg J. T. Ding M. Li W. (2022). Transcranial stimulation of alpha oscillations up-regulates the default mode network. PNAS 119:e2110868119. doi: 10.1073/pnas.2110868119, 34969856 PMC8740757

[ref26] Cole S. R. Voytek B. (2017). Brain oscillations and the importance of waveform shape. Trends Cogn. Sci. 21, 137–149. doi: 10.1016/j.tics.2016.12.008, 28063662

[ref27] Coppé J.-P. Desprez P.-Y. Krtolica A. Campisi J. (2010). The senescence-associated secretory phenotype: the dark side of tumor suppression. Annu. Rev. Pathol. 5, 99–118. doi: 10.1146/annurev-pathol-121808-102144, 20078217 PMC4166495

[ref28] Damasio A. R. (1994). Descartes’ Error: Emotion, Reason, and the Human Brain. New York: Putnam.

[ref29] Damasio A. R. (1999). The Feeling of what Happens: Body and Emotion in the Making of Consciousness. New York: Harcourt Brace.

[ref30] Damasio A. R. (2010). Self comes to mind: Constructing the Conscious brain. New York: Pantheon.

[ref31] Damasio A. Carvalho G. B. (2013). The nature of feelings: evolutionary and neurobiological origins. Nat. Rev. Neurosci. 14, 143–152. doi: 10.1038/nrn3403, 23329161

[ref32] Daniel T. D. (2018). Formal Dialectics. Newcastle Upon Tyne, UK: Cambridge Scholars Publishing.

[ref33] Davis H. E. McCorkell L. Vogel J. M. Topol E. J. (2023). Long COVID: major findings, mechanisms and recommendations. Nat. Rev. Microbiol. 21, 133–146. doi: 10.1038/s41579-022-00846-2, 36639608 PMC9839201

[ref34] Dennett D. C. (1991). Consciousness Explained. Boston: Little, Brown and Company.

[ref35] Djebbara Z. Fich L. B. Petrini L. Gramann K. (2021). Sensorimotor brain dynamics reflect architectural affordances. Sci. Rep. 11:2019. doi: 10.1038/s41598-021-82504-w, 31189596 PMC6642393

[ref36] Donoghue T. Haller M. Peterson E. J. Varma P. Sebastian P. Gao R. . (2020). Parameterizing neural power spectra into periodic and aperiodic components. Nat. Neurosci. 23, 1655–1665. doi: 10.1038/s41593-020-00744-x, 33230329 PMC8106550

[ref37] Edelman G. M. Gally J. A. (2001). Degeneracy and complexity in biological systems. PNAS 98, 13763–13768. doi: 10.1073/pnas.231499798, 11698650 PMC61115

[ref38] Erickson K. I. Voss M. W. Prakash R. S. Basak C. Szabo A. Chaddock L. . (2011). Exercise training increases size of hippocampus and improves memory. PNAS 108, 3017–3022. doi: 10.1073/pnas.1015950108, 21282661 PMC3041121

[ref39] Ermentrout G. B. Kopell N. (1990). Oscillator death in systems of coupled neural oscillators. SIAM J. Appl. Math. 50, 125–146.

[ref40] Finley A. J. Angus D. J. Knight E. van Reekum C. M. Lachman M. E. Davidson R. J. . (2024). Resting EEG periodic and aperiodic components predict cognitive decline over 10 years. J. Neurosci. 44:e1332232024. doi: 10.1523/JNEUROSCI.1332-23.2024, 38373849 PMC10977020

[ref41] Fluge Ø. Mella O. Bruland O. Risa K. Dyrstad S. E. Alme K. . (2016). Metabolic profiling indicates impaired pyruvate dehydrogenase function in myalgic encephalopathy/chronic fatigue syndrome. JCI Insight 1:e89376. doi: 10.1172/jci.insight.89376, 28018972 PMC5161229

[ref42] Fries P. (2005). A mechanism for cognitive dynamics: neuronal communication through neuronal coherence. Trends Cogn. Sci. 9, 474–480. doi: 10.1016/j.tics.2005.08.011, 16150631

[ref43] Fries P. (2015). Rhythms for cognition: communication through coherence. Neuron 88, 220–235. doi: 10.1016/j.neuron.2015.09.034, 26447583 PMC4605134

[ref44] Friston K. J. (2013). Life as we know it. J. R. Soc. Interface 10:20130475. doi: 10.1098/rsif.2013.0475, 23825119 PMC3730701

[ref45] Friston K. J. Fagerholm E. D. Zarghami T. S. Parr T. Hipólito I. Magrou L. . (2021). Parcels and particles: Markov blankets in the brain. Network Neurosci. 5, 211–251. doi: 10.1162/netn_a_00175, 33688613 PMC7935044

[ref46] Furness J. B. (2012). The enteric nervous system and neurogastroenterology. Nat. Rev. Gastroenterol. Hepatol. 9, 286–294. doi: 10.1038/nrgastro.2012.32, 22392290

[ref47] Gao R. Peterson E. J. Voytek B. (2017). Inferring synaptic excitation/inhibition balance from field potentials. NeuroImage 158, 70–78. doi: 10.1016/j.neuroimage.2017.06.078, 28676297

[ref48] Gedankien T. Tan R. J. Qasim S. E. Moore H. McDonagh D. Jacobs J. . (2023). Acetylcholine modulates the temporal dynamics of human theta oscillations during memory. Nat. Commun. 14:5283. doi: 10.1038/s41467-023-41025-y, 37648692 PMC10469188

[ref49] Glass L. (2001). Synchronization and rhythmic processes in physiology. Nature 410, 277–284. doi: 10.1038/35065745, 11258383

[ref50] Godlewska B. R. Sylvester A. L. Emir U. E. Sharpley A. L. Clarke W. T. Martens M. A. G. . (2024). Six-week supplementation with creatine in ME/CFS: a magnetic resonance spectroscopy feasibility study at 3 tesla. Nutrients 16:3308. doi: 10.3390/nu16193308, 39408275 PMC11478479

[ref51] Gonzales M. M. Garbarino V. R. Marques Zilli E. Petersen R. C. Kirkland J. L. Tchkonia T. . (2022). Senolytic therapy to modulate the progression of Alzheimer's disease (SToMP-AD): a pilot clinical trial. J. Prev Alzheimers Dis. 9, 22–29. doi: 10.14283/jpad.2021.62, 35098970 PMC8612719

[ref52] Gregoriou G. G. Gotts S. J. Zhou H. Desimone R. (2009). High-frequency, long-range coupling between prefrontal and visual cortex during attention. Science 324, 1207–1210. doi: 10.1126/science.1171402, 19478185 PMC2849291

[ref53] Gutkin B. S. Ermentrout G. B. Reyes A. D. (2005). Phase-response curves give the responses of neurons to transient inputs. J. Neurophysiol. 94, 1623–1635. doi: 10.1152/jn.00359.2004, 15829595

[ref54] Hacker C. D. Snyder A. Z. Pahwa M. Corbetta M. Leuthardt E. C. (2017). Frequency-specific electrophysiologic correlates of resting state fMRI networks. NeuroImage 149, 446–457. doi: 10.1016/j.neuroimage.2017.01.054, 28159686 PMC5745814

[ref55] Hahn G. Bujan A. F. Frégnac Y. Aertsen A. Kumar A. (2014). Communication through resonance in spiking neuronal networks. PLoS Comput. Biol. 10:e1003811. doi: 10.1371/journal.pcbi.1003811, 25165853 PMC4148205

[ref56] Hampshire A. Azor A. Atchison C. Trender W. Hellyer P. J. Giunchiglia V. . (2024). Cognition and memory after Covid-19 in a large community sample. N. Engl. J. Med. 390, 806–818. doi: 10.1056/NEJMoa2311330, 38416429 PMC7615803

[ref57] Harmony T. (2013). The functional significance of delta oscillations in cognitive processing. Front. Integr. Neurosci. 7:83. doi: 10.3389/fnint.2013.00083, 24367301 PMC3851789

[ref58] Hasselmo M. E. Bodelón C. Wyble B. P. (2002). A proposed function for hippocampal theta rhythm: separate phases of encoding and retrieval enhance reversal of prior learning. Neural Comput. 14, 793–817. doi: 10.1162/089976602317318965, 11936962

[ref59] He B. J. Zempel J. M. Snyder A. Z. Raichle M. E. (2010). The temporal structures and functional significance of scale-free brain activity. Neuron 66, 353–369. doi: 10.1016/j.neuron.2010.04.020, 20471349 PMC2878725

[ref60] Helfrich R. F. Knight R. T. (2016). Oscillatory dynamics of prefrontal cognitive control. Trends Cogn. Sci. 20, 916–930. doi: 10.1016/j.tics.2016.09.007, 27743685 PMC5127407

[ref61] Hille B. (2001). Ion Channels of Excitable Membranes. 3rd Edn. Sunderland, MA, USA Sinauer Associates.

[ref62] Hillebrand A. Tewarie P. van Dellen E. Yu M. Carbo E. W. S. Douw L. . (2016). Direction of information flow in large-scale resting-state networks is frequency-dependent. PNAS 113, 3867–3872. doi: 10.1073/pnas.1515657113, 27001844 PMC4833227

[ref63] Hipólito I. Ramstead M. J. D. Convertino L. Bhat A. Friston K. J. Parr T. (2021). Markov blankets in the brain. Neurosci. Biobehav. Rev. 125, 88–97. doi: 10.1016/j.neubiorev.2021.02.003, 33607182 PMC8373616

[ref64] Holroyd C. B. (2025). The controllosphere: the neural origin of cognitive effort. Psychol. Rev. 132, 603–631. doi: 10.1037/rev0000467, 38358716

[ref65] Institute of Medicine (2015). Beyond Myalgic Encephalomyelitis/Chronic Fatigue Syndrome: Redefining an Illness. Washington: National Academies Press. 25695122

[ref66] Jékely G. Keijzer F. Godfrey-Smith P. (2015). An option space for early neural evolution. Philos. Trans. R. Soc. B 370:20150181. doi: 10.1098/rstb.2015.0181, 26554049 PMC4650133

[ref67] Kawasaki M. Kitajo K. Yamaguchi Y. (2010). Dynamic links between theta executive functions and alpha storage buffers in auditory and visual working memory. Eur. J. Neurosci. 31, 1683–1689. doi: 10.1111/j.1460-9568.2010.07217.x, 20525081 PMC2878597

[ref68] Kelso J. A. S. (1995). Dynamic Patterns: The Self-Organization of brain and Behavior. Cambridge, MA, USA MIT Press.

[ref69] Kelso J. A. S. (2012). Multistability and metastability: understanding dynamic coordination in the brain. Philos. Trans. R. Soc. B 367, 906–918. doi: 10.1098/rstb.2011.0351, 22371613 PMC3282307

[ref70] Kelso J. A. S. (2021). Unifying large- and small-scale theories of coordination. Entropy 23:537. doi: 10.3390/e23050537, 33925736 PMC8146522

[ref71] Kirchhoff M. Parr T. Palacios E. Friston K. Kiverstein J. (2018). The Markov blankets of life: autonomy, active inference and the free energy principle. J. R. Soc. Interface 15:20170792. doi: 10.1098/rsif.2017.0792, 29343629 PMC5805980

[ref72] Knyazev G. G. Slobodskoj-Plusnin J. Y. Bocharov A. V. Pylkova L. V. (2011). The default mode network and EEG alpha oscillations: an independent component analysis. Brain Res. 1402, 67–79. doi: 10.1016/j.brainres.2011.05.052, 21683942

[ref73] Koller D. P. Schirner M. Ritter P. (2024). Human connectome topology directs cortical traveling waves and shapes frequency gradients. Nat. Commun. 15:3570. doi: 10.1038/s41467-024-47860-x, 38670965 PMC11053146

[ref74] Kopčanová M. Tait L. Donoghue T. Stothart G. Smith L. Flores-Sandoval A. A. . (2024). Resting-state EEG signatures of Alzheimer's disease are driven by periodic but not aperiodic changes. Neurobiol. Dis. 190:106380. doi: 10.1016/j.nbd.2023.106380, 38114048

[ref75] Lachaux J.-P. Rodriguez E. Martinerie J. Varela F. J. (1999). Measuring phase synchrony in brain signals. Hum. Brain Mapp. 8, 194–208. 10619414 10.1002/(SICI)1097-0193(1999)8:4<194::AID-HBM4>3.0.CO;2-C PMC6873296

[ref76] Lange M. Joly F. Vardy J. Ahles T. Dubois M. Tron L. . (2019). Cancer-related cognitive impairment: an update on state of the art, detection, and management strategies in cancer survivors. Ann. Oncol. 30, 1925–1940. doi: 10.1093/annonc/mdz410, 31617564 PMC8109411

[ref77] Lennie P. (2003). The cost of cortical computation. Curr. Biol. 13, 493–497. doi: 10.1016/s0960-9822(03)00135-0 12646132

[ref78] Levin M. (2022). Technological approach to mind everywhere: an experimentally-grounded framework for understanding diverse bodies and minds. Front. Syst. Neurosci. 16:768201. doi: 10.3389/fnsys.2022.768201, 35401131 PMC8988303

[ref79] Levy W. B. Calvert V. G. (2021). Communication consumes 35 times more energy than computation in the human cortex. PNAS 118:e2008173118. doi: 10.1073/pnas.2008173118, 33906943 PMC8106317

[ref80] Lisman J. (2005). The theta/gamma discrete phase code occurring during the hippocampal phase precession may be a more general brain coding scheme. Hippocampus 15, 913–922. doi: 10.1002/hipo.20121, 16161035

[ref81] Lu R. Dermody N. Duncan J. Woolgar A. (2024). Aperiodic and oscillatory systems underpinning human domain-general cognition. Commun. Biol. 7:1643. doi: 10.1038/s42003-024-07397-7, 39695307 PMC11655660

[ref82] Lundqvist M. Rose J. Herman P. Brincat S. L. Buschman T. J. Miller E. K. (2016). Gamma and beta bursts underlie working memory. Neuron 90, 152–164. doi: 10.1016/j.neuron.2016.02.028, 26996084 PMC5220584

[ref83] Ma Y. Brown J. A. Chen C. Ding M. Wu W. Li W. (2025). Alpha-frequency stimulation enhances synchronization of alpha oscillations with default mode network connectivity. eNeuro 12:ENEURO.0449-24.2025. doi: 10.1523/eneuro.0449-24.2025, 40068873 PMC11927933

[ref84] Mahjoory K. Schoffelen J.-M. Keitel A. Gross J. (2020). The frequency gradient of human resting-state brain oscillations follows cortical hierarchies. eLife 9:e53715. doi: 10.7554/elife.53715, 32820722 PMC7476753

[ref85] Mantini D. Perrucci M. G. Del Gratta C. Romani G. L. Corbetta M. (2007). Electrophysiological signatures of resting state networks in the human brain. PNAS 104, 13170–13175. doi: 10.1073/pnas.0700668104, 17670949 PMC1941820

[ref86] Marder E. (2012). Neuromodulation of neuronal circuits: Back to the future. Neuron 76, 1–11. doi: 10.1016/j.neuron.2012.09.010, 23040802 PMC3482119

[ref87] Mashour G. A. Hudetz A. G. (2018). Neural correlates of unconsciousness in large-scale brain networks. Trends Neurosci. 41, 150–160. doi: 10.1016/j.tins.2018.01.003, 29409683 PMC5835202

[ref88] McMillen P. Levin M. (2024). Collective intelligence: a unifying concept for integrating biology across scales and substrates. Commun. Biol. 7:378. doi: 10.1038/s42003-024-06037-4, 38548821 PMC10978875

[ref89] Miyake A. Friedman N. P. Emerson M. J. Witzki A. H. Howerter A. Wager T. D. (2000). The unity and diversity of executive functions and their contributions to complex “frontal lobe” tasks. Cogn. Psychol. 41, 49–100. doi: 10.1006/cogp.1999.0734, 10945922

[ref90] Mlinarič T. Spruyt L. Khachatryan E. Wittevrongel B. Reinartz M. Van Laere K. . (2026). Early aperiodic EEG changes in preclinical and prodromal Alzheimer's disease. Alzheimer's Res. Ther. 18:28. doi: 10.1186/s13195-026-01953-5, 41501948 PMC12882448

[ref91] Mukamel E. A. Pirondini E. Babadi B. Wong K. F. K. Pierce E. T. Harrell P. G. . (2014). A transition in brain state during propofol-induced unconsciousness. J. Neurosci. 34, 839–845. doi: 10.1523/jneurosci.5813-12.2014, 24431442 PMC3891963

[ref92] Muller L. Chavane F. Reynolds J. Sejnowski T. J. (2018). Cortical travelling waves: mechanisms and computational principles. Nat. Rev. Neurosci. 19, 255–268. doi: 10.1038/nrn.2018.20, 29563572 PMC5933075

[ref93] Nelson G. Wordsworth J. Wang C. Jurk D. Lawless C. Martin-Ruiz C. . (2012). A senescent cell bystander effect: senescence-induced senescence. Aging Cell 11, 345–349. doi: 10.1111/j.1474-9726.2012.00795.x, 22321662 PMC3488292

[ref94] Neubauer S. Horn M. Cramer M. Harre K. Newell J. B. Peters W. . (1997). Myocardial phosphocreatine-to-ATP ratio is a predictor of mortality in patients with dilated cardiomyopathy. Circulation 96, 2190–2196. 9337189 10.1161/01.cir.96.7.2190

[ref95] Nicholson D. J. (2019). Is the cell really a machine? J. Theor. Biol. 477, 108–126. doi: 10.1016/j.jtbi.2019.06.002 31173758

[ref96] Nicholson A. A. Densmore M. Frewen P. A. Neufeld R. W. J. Théberge J. Jetly R. . (2023). Homeostatic normalization of alpha brain rhythms within the default-mode network and reduced symptoms in PTSD following a randomized controlled trial of EEG neurofeedback. Brain Commun. 5:fcad068. doi: 10.1093/braincomms/fcad068, 37065092 PMC10090479

[ref97] O’Keefe J. Recce M. L. (1993). Phase relationship between hippocampal place units and the EEG theta rhythm. Hippocampus 3, 317–330. 8353611 10.1002/hipo.450030307

[ref98] Ogrodnik M. Evans S. A. Fielder E. Victorelli S. Kruger P. Salmonowicz H. . (2021). Whole-body senescent cell clearance alleviates age-related brain inflammation and cognitive impairment in mice. Aging Cell 20:e13296. doi: 10.1111/acel.13296, 33470505 PMC7884042

[ref99] Oyler-Yaniv A. Oyler-Yaniv J. Whitlock B. M. Liu Z. Germain R. N. Huse M. . (2017). A tunable diffusion-consumption mechanism of cytokine propagation enables plasticity in cell-to-cell communication in the immune system. Immunity 46, 609–620. doi: 10.1016/j.immuni.2017.03.011, 28389069 PMC5442880

[ref100] Palacios E. R. Razi A. Parr T. Kirchhoff M. Friston K. (2020). On Markov blankets and hierarchical self-organisation. J. Theor. Biol. 486:110089. doi: 10.1016/j.jtbi.2019.110089, 31756340 PMC7284313

[ref101] Palmigiano A. Geisel T. Wolf F. Battaglia D. (2017). Flexible information routing by transient synchrony. Nat. Neurosci. 20, 1014–1022. doi: 10.1038/nn.4569, 28530664

[ref102] Palva S. Palva J. M. (2007). New vistas for alpha-frequency band oscillations. Trends Neurosci. 30, 150–158. doi: 10.1016/j.tins.2007.02.001, 17307258

[ref103] Palva S. Palva J. M. (2011). Functional roles of alpha-band phase synchronization in local and large-scale cortical networks. Front. Psychol. 2:204. doi: 10.3389/fpsyg.2011.00204, 21922012 PMC3166799

[ref104] Purdon P. L. Pierce E. T. Mukamel E. A. Prerau M. J. Walsh J. L. Wong K. F. K. . (2013). Electroencephalogram signatures of loss and recovery of consciousness from propofol. PNAS 110, E1142–E1151. doi: 10.1073/pnas.1221180110, 23487781 PMC3607036

[ref105] Ruuskanen S. Avendano-Diaz J. C. Liljeström M. Parkkonen L. (2026). Frequency-resolved cortical functional connectivity across the adult lifespan. Hum. Brain Mapp. 47:e70484. doi: 10.1002/hbm.70484, 41755554 PMC12946715

[ref106] Saby J. N. Marshall P. J. (2012). The utility of EEG band power analysis in the study of infancy and early childhood. Dev. Neuropsychol. 37, 253–273. doi: 10.1080/87565641.2011.614663, 22545661 PMC3347767

[ref107] Sadaghiani S. Scheeringa R. Lehongre K. Morillon B. Giraud A. L. Kleinschmidt A. (2010). Intrinsic connectivity networks, alpha oscillations, and tonic alertness: a simultaneous EEG/fMRI study. J. Neurosci. 30, 10243–10250. doi: 10.1523/jneurosci.1004-10.2010, 20668207 PMC6633365

[ref108] Sauseng P. Klimesch W. Schabus M. Doppelmayr M. (2005). Fronto-parietal EEG coherence in theta and upper alpha reflect central executive functions. Int. J. Psychophysiol. 57, 97–103. doi: 10.1016/j.ijpsycho.2005.03.018, 15967528

[ref109] Scheffer-Teixeira R. Tort A. B. L. (2016). On cross-frequency phase-phase coupling between theta and gamma oscillations in the hippocampus. eLife 5:e20515. doi: 10.7554/elife.20515, 27925581 PMC5199196

[ref110] Schneider M. Broggini A. C. Dann B. Tzanou A. Uran C. Sheshadri S. . (2021). A mechanism for inter-areal coherence through communication based on connectivity and oscillatory power. Neuron 109, 4050–4067.e12. doi: 10.1016/j.neuron.2021.09.037, 34637706 PMC8691951

[ref111] Schreiber T. (2000). Measuring information transfer. Phys. Rev. Lett. 85, 461–464. 10991308 10.1103/PhysRevLett.85.461

[ref112] Silva-Passadouro B. Tamasauskas A. Grieve S. Chalder T. Bhatt N. (2024). A systematic review of EEG findings in chronic fatigue syndrome/myalgic encephalomyelitis. Clin. Neurophysiol. 163, 209–222. doi: 10.1016/j.clinph.2024.04.019, 38772083

[ref113] Singh A. B. Harris R. C. (2005). Autocrine, paracrine and juxtacrine signaling by EGFR ligands. Cell. Signal. 17, 1183–1193. doi: 10.1016/j.cellsig.2005.03.026, 15982853

[ref114] Slankamenac J. Ranisavljev M. Todorovic N. Ostojic J. Stajer V. Ostojic S. M. (2023). Effects of six-month creatine supplementation on patient- and clinician-reported outcomes in post-COVID-19 fatigue syndrome. Food Sci. Nutr. 11, 6899–6906. doi: 10.1002/fsn3.3597, 37970399 PMC10630839

[ref115] Spitzer B. Haegens S. (2017). Beyond the status quo: a role for beta oscillations in endogenous content (re)activation. eNeuro 4:ENEURO.0170-17.2017. doi: 10.1523/eneuro.0170-17.2017, 28785729 PMC5539431

[ref116] Stoffregen T. A. Wagman J. B. (2025). Higher order affordances. Psychon. Bull. Rev. 32, 1–30. doi: 10.3758/s13423-024-02535-y, 38944659

[ref117] Taylor H. P. Huynh K. M. Thung K.-H. Lin G. Lyu W. Lin W. . (2026). Functional hierarchy of the human neocortex across the lifespan. Nature 652, 955–964. doi: 10.1038/s41586-026-10219-x, 41882352 PMC13102691

[ref118] Thibault R. T. Lifshitz M. Raz A. (2017). Neurofeedback or neuroplacebo? Brain 140, 862–864. doi: 10.1093/brain/awx033 28375458

[ref119] Thibault R. T. MacPherson A. Lifshitz M. Roth R. R. Raz A. (2018). Neurofeedback with fMRI: a critical systematic review. NeuroImage 172, 786–807. doi: 10.1016/j.neuroimage.2017.12.071, 29288868

[ref120] Thompson E. (2004). Life and mind: from autopoiesis to neurophenomenology. A tribute to Francisco Varela. Phenomenol. Cogn. Sci. 3, 381–398. doi: 10.1023/B:PHEN.0000048936.73339.dd

[ref121] Tort A. B. L. Komorowski R. Eichenbaum H. Kopell N. (2010). Measuring phase-amplitude coupling between neuronal oscillations of different frequencies. J. Neurophysiol. 104, 1195–1210. doi: 10.1152/jn.00106.2010, 20463205 PMC2941206

[ref122] Turner C. E. Byblow W. D. Gant N. (2015). Creatine supplementation enhances corticomotor excitability and cognitive performance during oxygen deprivation. J. Neurosci. 35, 1773–1780. doi: 10.1523/jneurosci.3113-14.2015, 25632150 PMC6795258

[ref123] Uhlhaas P. J. Roux F. Rodriguez E. Rotarska-Jagiela A. Singer W. (2010). Neural synchrony and the development of cortical networks. Trends Cogn. Sci. 14, 72–80. doi: 10.1016/j.tics.2009.12.002, 20080054

[ref124] Varela F. J. Thompson E. Rosch E. (1991). The Embodied Mind: Cognitive Science and Human Experience. Cambridge, MA MIT Press.

[ref125] Vinck M. Oostenveld R. van Wingerden M. Battaglia F. Pennartz C. M. A. (2011). An improved index of phase-synchronization for electrophysiological data in the presence of volume-conduction, noise and sample-size bias. NeuroImage 55, 1548–1565. doi: 10.1016/j.neuroimage.2011.01.055 21276857

[ref126] Voytek B. Kramer M. A. Case J. Lepage K. Q. Tempesta Z. R. Knight R. T. . (2015). Age-related changes in 1/f neural electrophysiological noise. J. Neurosci. 35, 13257–13265. doi: 10.1523/jneurosci.2332-14.2015, 26400953 PMC4579381

[ref127] Wang P.-Y. Ma J. Kim Y. C. Son A. Y. Syed A. M. Liu C. . (2023). WASF3 disrupts mitochondrial respiration and may mediate exercise intolerance in ME/CFS. PNAS 120:e2302738120. doi: 10.1073/pnas.2302738120, 37579159 PMC10450651

[ref128] Weiss E. Kann M. Wang Q. (2023). Neuromodulation of neural oscillations in health and disease. Biology 12:371. doi: 10.3390/biology12030371, 36979063 PMC10045166

[ref129] Williams P. L. Beer R. D. (2010). Nonnegative decomposition of multivariate information. arXiv. doi: 10.48550/arXiv.1004.2515

[ref130] Yu A. J. Dayan P. (2005). Uncertainty, neuromodulation, and attention. Neuron 46, 681–692. doi: 10.1016/j.neuron.2005.04.026, 15944135

[ref131] Zhang Y. Karadas M. Liu J. Gu X. Voroeslakos M. Li Y. . (2024). Interaction of acetylcholine and oxytocin neuromodulation in the hippocampus. Neuron 112, 1862–1875.e5. doi: 10.1016/j.neuron.2024.02.021, 38537642 PMC11156550

[ref132] Zhang H. Watrous A. J. Patel A. Jacobs J. (2018). Theta and alpha oscillations are traveling waves in the human neocortex. Neuron 98, 1269–1281.e4. doi: 10.1016/j.neuron.2018.05.019, 29887341 PMC6534129

[ref133] Zinn M. A. Zinn M. L. Valencia I. Jason L. A. Montoya J. G. (2018). Cortical hypoactivation during resting EEG suggests central nervous system pathology in patients with chronic fatigue syndrome. Biol. Psychol. 136, 87–99. doi: 10.1016/j.biopsycho.2018.05.016 29802861 PMC6064389

